# Supplementary material for: Gender differences in unpaid care work and psychological distress in the UK Covid-19 lockdown
Source: PLoS One. 2021 Mar 4;16(3):e0247959. doi: 10.1371/journal.pone.0247959 (PMC7932161; doi:10.1371/journal.pone.0247959)
Supplement: S1 Table — (DOCX) [file pone.0247959.s001.docx]

**S1 Table. Sample types for analysis**

| **Unpaid care type** | **Exposure variables** | **Eligible sample** | **Eligible sample N** | **Complete case N** | **Source of missing data ^a^**  **April (May)** |
| --- | --- | --- | --- | --- | --- |
| Housework | Own housework hours | Every participant | April 17452;  May 14811 | April 13218; May 12472 | 9.6% (2.5%) due to missing GHQ in lockdown  Further 2.2% (2%) due to missing in housework  Further 14.3% (11.9%) due to missing in covariates |
|  | Women’s share of housework within couples | Participants living as a couple in Covid survey & able to link together from wave 9 identifier ^b^ | April 7270;  May 6137 | April 7009; May 5656 | 3.7% (0.9%) due to missing GHQ in lockdown  Further 4.4 % (14.6%) due to missing in relative housework  Further 5.7% (4.9%) due to missing in covariates |
| Childcare  /homeschooling | Own childcare/ homeschooling hours per week | Parents who have one or more children aged 18 or under in the household | April 6166; May 4615 | April 4174; May 3179 | 14.6% (3%) due to missing GHQ in lockdown  Further 2.3% (2.4%) due to missing in childcare  Further 18.7% (14.9%) due to missing in covariates |
|  | Women’s share of childcare/homeschooling within couples | Couple parents who have one or more children aged 18 or under in the household & able to link together from wave 9 identifier ^b^ | April 2563;  May 2068 | April 1731; May 1551 | 6.2% (1.3%) due to missing GHQ in lockdown  Further 21.7% (29.1%) due to missing in relative childcare  Further 7.8% (5.7%) due to missing in covariates |
| Employment adjustment due to childcare  /homeschooling | Own employment hour adjustment | Parents working more than zero hours in January or February | April N/A;  May 3733 | April N/A;  May 2990 | (2.7%) due to missing GHQ in lockdown  Further (3.8 %) due to missing in employment adjustment  Further (14.5%) due to missing in covariates |
|  | Own employment pattern adjustment | Parents working more than zero hours in January or February | April N/A;  May 3733 | April N/A;  May 2983 | (2.7%) due to missing GHQ in lockdown  Further (3.9 %) due to missing in employment adjustment  Further (14.5%) due to missing in covariates |
|  | Employment hour adjustment within couples | Couple parents working more than zero hours in January or February & able to link together from wave 9 identifier ^b^ | April N/A;  May 1800 | April N/A;  May 1572 | (<1%) due to missing GHQ in lockdown  (12%) due to missing in couple level employment adjustment |
|  | Employment pattern adjustment within couples | Couple parents working more than zero hours in January or February & able to link together from wave 9 identifier ^b^ | April N/A;  May 1800 | April N/A;  May 1572 | (<1%) due to missing GHQ in lockdown  (12%) due to missing in couple level employment adjustment |

^a^ % missing in May are shown in brackets.

^b^ Covid survey only asked whether participants were living with a couple, so wave 9 identifier was used to identify the members in a couple.
