# Supplementary material for: Gender differences in unpaid care work and psychological distress in the UK Covid-19 lockdown
Source: PLoS One. 2021 Mar 4;16(3):e0247959. doi: 10.1371/journal.pone.0247959 (PMC7932161; doi:10.1371/journal.pone.0247959)
Supplement: S2 Table — (DOCX) [file pone.0247959.s002.docx]

**S2 Table. Gender differences in individual-level unpaid care work after adjusting for demographic differences in April wave.**

|  | **Unadjusted model** | | **Adjusted model without employment^a^** | | **Full model^b^** | |
| --- | --- | --- | --- | --- | --- | --- |
|  | **Coefficient^c^**  **(95%CI)** | **Men; Women^d^** | **Coefficient^c^**  **(95%CI)** | **Men; Women^d^** | **Coefficient^c^**  **(95%CI)** | **Men; Women^d^** |
| Housework hours per week  (n=13,218) | **5.17**  (4.83, 5.51) | 9.88; 15.05 | **5.25**  (4.92,5.59) | 9.83; 15.09 | **5.06**  (4.71, 5.41) | 9.95; 15.01 |
| Childcare/ homeschooling hours per week  (n=4,174) | **8.93**  (7.24,10.62) | 12.25; 21.18 | **7.22**  (5.63, 8.80) | 13.31;20.53 | **5.96**  (4.25, 7.67) | 14.09; 20.05 |

^a^ Model adjusted for age, ethnicity, living with a partner, number of children in the household by children’s age group, qualifications, occupational class, and baseline GHQ.

^b^ Model further adjusted for baseline working hours.

^c^ Coefficient of gender in the linear regression model between gender and unpaid care work (housework/ childcare).

^d^ Average marginal values of unpaid care work hours per week predicted from the regression model.
