# Supplementary material for: Gender differences in unpaid care work and psychological distress in the UK Covid-19 lockdown
Source: PLoS One. 2021 Mar 4;16(3):e0247959. doi: 10.1371/journal.pone.0247959 (PMC7932161; doi:10.1371/journal.pone.0247959)
Supplement: S3 Table — (DOCX) [file pone.0247959.s003.docx]

**S3 Table. Gender differences in individual-level unpaid care work after adjusting for demographic differences in May wave.**

|  | **Unadjusted model** | | **Adjusted model without employment^a^** | | **Full model^b^** | |
| --- | --- | --- | --- | --- | --- | --- |
|  | **Coefficient**  **/OR**  **(95%CI)** | **Men; Women^c^** | **Coefficient**  **/OR**  **(95%CI)** | **Men; Women^c^** | **Coefficient**  **/OR**  **(95%CI)** | **Men; Women^c^** |
| Housework hours per week  (n=12,472) | 5.52 ^d^  (5.17, 5.87) | 9.52; 15.04 | 5.70 ^d^  (5.35,6.05) | 9.41; 15.11 | 5.48 ^d^  (5.12, 5.84) | 9.54; 15.02 |
| Childcare/ homeschooling hours per week  (n=3,719) | 9.19 ^d^  (7.40, 10.98) | 11.57; 20.76 | 8.03 ^d^  (6.29, 9.76) | 12.29; 20.32 | 6.31 ^d^  (4.44, 8.18) | 13.35; 19.66 |
| Reduce employment hours due to  childcare/ homeschooling (n=2,990) | 1.58 ^e^  (1.26, 1.96) | 11.3%; 16.7% | 1.69 ^e^  (1.34, 2.15) | 11.3%; 17.1% | 1.64 ^e^  (1.26, 2.13) | 11.5%; 16.9% |
| Adapted work patterns due to  childcare/ homeschooling (n=2,983) | 1.35  (1.16, 1.58) | 30.1%; 36.9% | 1.52  (1.27, 1.83) | 29.7%; 37.2% | 1.66 ^e^  (1.36, 2.04) | 28.9%; 37.9% |

^a^ Model adjusted for age, ethnicity, living with a partner, number of children in the household by children’s age group, qualifications, occupational class, and baseline GHQ.

^b^ Model further adjusted for baseline working hours.

^c^ Average marginal values of unpaid care work hours or % of work adaption predicted from the regression model.

^d^ Coefficient of gender in the linear regression model between gender and unpaid care work (housework/ childcare).

^e^ OR of gender the logistic regression model between gender and unpaid care work (work adaption).
