# Supplementary material for: Gender differences in unpaid care work and psychological distress in the UK Covid-19 lockdown
Source: PLoS One. 2021 Mar 4;16(3):e0247959. doi: 10.1371/journal.pone.0247959 (PMC7932161; doi:10.1371/journal.pone.0247959)
Supplement: S4 Table — (DOCX) [file pone.0247959.s004.docx]

**S4 Table. Actor-partner effects in the association between unpaid care and psychological distress amongst couples.**

|  | **Men April** | | **Women April** | | **Men May** | | **Women May** | |
| --- | --- | --- | --- | --- | --- | --- | --- | --- |
|  | **Coefficient**  **(95%CI)** | ***p*-value** | **Coefficient**  **(95%CI)** | ***p*-value** | **Coefficient**  **(95%CI)** | ***p*-value** | **Coefficient**  **(95%CI)** | ***p*-value** |
| **Housework** |  |  |  |  |  |  |  |  |
| Own hours | 0.006  (-0.029, 0.041) | 0.744 | 0.010  (-0.021, 0.04) | 0.525 | -0.0005  (-0.041, 0.040) | 0.982 | 0.009  (-0.024, 0.041) | 0.603 |
| Partner’s hours | 0.006  (-0.027, 0.038) | 0.736 | -0.011  (-0.063, 0.040) | 0.666 | -0.009  (-0.040, 0.023) | 0.593 | 0.042  (-0.001, 0.085) | 0.054 |
| **Childcare/**  **homeschooling** |  |  |  |  |  |  |  |  |
| Own hours | 0.009  (-0.021, 0.039) | 0.566 | 0.018  (-0.002,0.038) | 0.072 | 0.013  (-0.018, 0.044) | 0.415 | **0.030**  (0.012, 0.047) | 0.001 |
| Partner’s hours | 0.006  (-0.009, 0.021) | 0.467 | 0.002  (-0.023, 0.027) | 0.905 | 0.010  (-0.006, 0.025) | 0.235 | -0.008  (-0.049, 0.034) | 0.719 |

Results of S4 Table show that women’s own childcare/ homeschooling hours were associated with a higher level of distress, and the coefficient was 0.018 (95% CI: -0.002,0.038; p=0.072) at April wave and was 0.030 (95% CI: 0.012, 0.047; p=0.001) at May wave. No significant result of partner effect (i.e., partner’s hours) was found in addition to the actor effect (i.e., own hours). However, there was a large coefficient (0.042) for partner’s housework hours in May, although it was not statically significant at the 5% level (p=0.054). This positive coefficient suggests that higher partner’s housework hours may be associated with women’s higher distress in May. This result is surprising. However, it is possible that women who increased more psychological distress at April may feel less able to do the same amount of housework in May, and thus, their partner has stepped in doing more housework in May. To test this, we further adjusted the GHQ score at April in the model (results are not shown in tables), and the association with the partner’s housework hours has reduced to 0.012 (p=0.557).
